# Femoral neck osteotomy guide for total hip arthroplasty

**DOI:** 10.1186/s12893-015-0015-3

**Published:** 2015-03-18

**Authors:** Lei Yang, Zhanle Zheng, Wei Chen, Juan Wang, Yingze Zhang

**Affiliations:** Department of Orthopaedic Surgery and Key Biomechanical Laboratory of Orthopaedics of Hebei Province, The Third Hospital of Hebei Medical University, Shijiazhuang, 050051 China

**Keywords:** Guide, Femoral neck osteotomy, Osteotomy guide, Total hip arthroplasty, Leg length discrepancy

## Abstract

**Background:**

Total hip arthroplasty (THA) is a common last-resort treatment for hip disease, but postoperative patients often complain of discrepancies in leg length. This study introduces a device designed to increase the precision of the femoral neck osteotomy and reduce the incidence of leg length discrepancy.

**Methods:**

Forty-eight patients undergoing THA were divided into two groups, with and without the use of the femoral osteotomy guide. All operations were performed through a posterolateral approach. Differences in leg length were recorded before and after the operation. Measurements were also made to compare the preoperative plan with the actual amount of bone removed.

**Results:**

The mean average difference in femoral neck resection height was 0.84 mm when using the osteotomy guide and 1.69 mm without the guide. Discrepancies in postoperative leg length were 5.45 mm and 13.37 mm in the groups with and without the guide, respectively.

**Conclusion:**

The femoral neck osteotomy guide is an effectively auxiliary tool for increasing the accuracy of bone resection in arthroplasty using the posterolateral approach.

**Trial registration:**

ChiCTR-OOC-15005904; date: 2015-01-30

## Background

Total hip arthroplasty (THA) is the most effective surgical approach for reducing pain and preserving function in heavily degraded joints [1]. Leg length discrepancy (LLD) of 2 - 3 cm is critical for the clinical outcomes. Older patients may have problems with LLD as small as 2 cm. But Clark et al reported patients complained when LLD was ≥ 1 cm [2]. The procedure requires removing a certain degree of bone block from the femoral head and neck and often results in a post-surgical LLD, a problem that surgeons have long-sought to rectify. The position of the femoral neck osteotomy is one of the crucial factors in avoiding LLD [3]. To fit the hip joint prostheses during THA, the planes and angles of the femoral neck osteotomy require stringent control. Typically, a surgeon can only rely on his or her own experience with the procedure, which can often lead to errors between the planned fitting and the actual surgical alignment. This practice is not conducive to patient rehabilitation because it not only extends the operative time but also affects the results of the operation. To improve the accuracy of the femoral neck osteotomy, we designed a femoral neck osteotomy guide and compared its performance against a commonly-used unguided osteotomy technique. This study aims to introduce and assess the reliability of the guide designed for osteotomy in THA.

## Methods

### Structure of the femoral neck osteotomy guide

**Figure 1:**
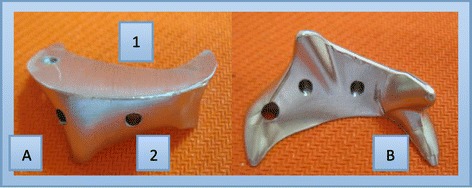
**Femoral Neck Osteotomy Guide A: anterior view, B: posterior view.** The guide device comprising two parts, the upper oriented platform (1) and the lower locating seat, which is attached to the top of the lesser femoral trochanter (2).

The femoral neck osteotomy guide (Figure 1) consists of two parts: the upper part is an oriented platform that guides the direction of the femoral neck osteotomy perpendicular to the long axis of femoral neck, and the lower part locates on the top of the lesser trochanter of the femur. There are two fixed screw holes in the locating seat, which are used to fix the device on the femoral neck. The guides were divided into left and right groups, and a series of the guides was designed according to the osteotomy height from 10 mm to 15 mm for each group. The series contains six models with a 1 mm interval difference between each two adjacent models. The different sizes of the osteotomy guides were selected according to preoperative templates on X-ray films, such as a 10-mm guide for a 10 mm osteotomy height, and the like. First, a model was made and modified on femur specimens using a self-curing denture acrylic. The model was then tested and adjusted on 128 femur specimens to obtain a suitable model form for a variety of femoral neck configurations. Finally the guide was made from titanium with a ratio of 1:1 according to the model. The guide was suitable for THA through a posterolateral approach. Before surgery, the device was sterilised with low temperature hydrogen peroxide gas plasma sterilisation.

### Patients

**Figure 2:**
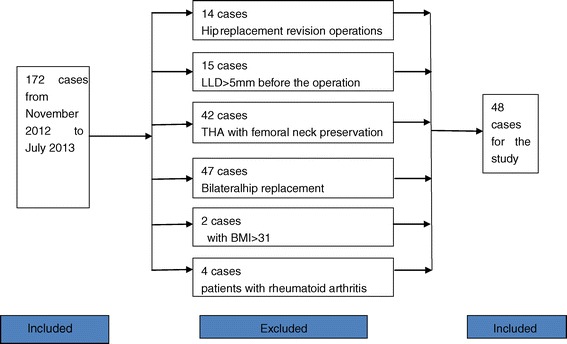
**Flowchart illustrates the patients selected for study.**

This study aimed to investigate the precision of the guide for THA. Between November 2012 and July 2013, 48 patients in need of femoral neck osteotomy were included in our study (Figure 2). Inclusion criteria were: primary hip arthroplasty with limb-length discrepancy less than 5 mm. In these patients, only one side required hip replacement while the other side was normal. Exclusion criteria were: preoperative LLD > 5 mm by tape measurement, total hip arthroplasty with femoral neck preservation and a history of previous hip surgery. Patients were also excluded from the study if they had obesity, rheumatoid arthritis, scoliosis, pelvic obliquity, or a limp caused by cerebral thrombosis, cerebral palsy and other causes.

The selected patients were randomly assigned into the two groups. Twenty-four patients were operated on with the use of the guide (group I) and 24 without the guide (group II). All operations were performed by the same experienced surgeon and measurements were made by a member of the research team. The surgical team consisted of surgeons with more than 10 years of experience in hip replacement. Data on age, gender, operation side, an etiology of surgical indications, femoral neck height, leg length and operative time were collected for both groups.

### Surgical techniques

All patients were admitted to the hospital 3-5 days before the surgery. Leg length was calculated and recorded by tape measure with the patients in a supine position before and after the THA surgery. The length of the lower limbs was measured between the anterior superior iliac spine (ASIS) and the medial malleolus [4]. Preoperative radiographs were taken from an anteroposterior view of the pelvis with both femurs internally rotated approximately 15° as required for assessment before THA. The templates were used to determine the height of the femoral neck osteotomy and the potential correct sizes for both the acetabular and femoral components of the prostheses. After surgery, the heights of the osteotomies were measured from the lesser trochanter to the cuneiform plane on the X-ray films using the same parameters.

**Figure 3:**
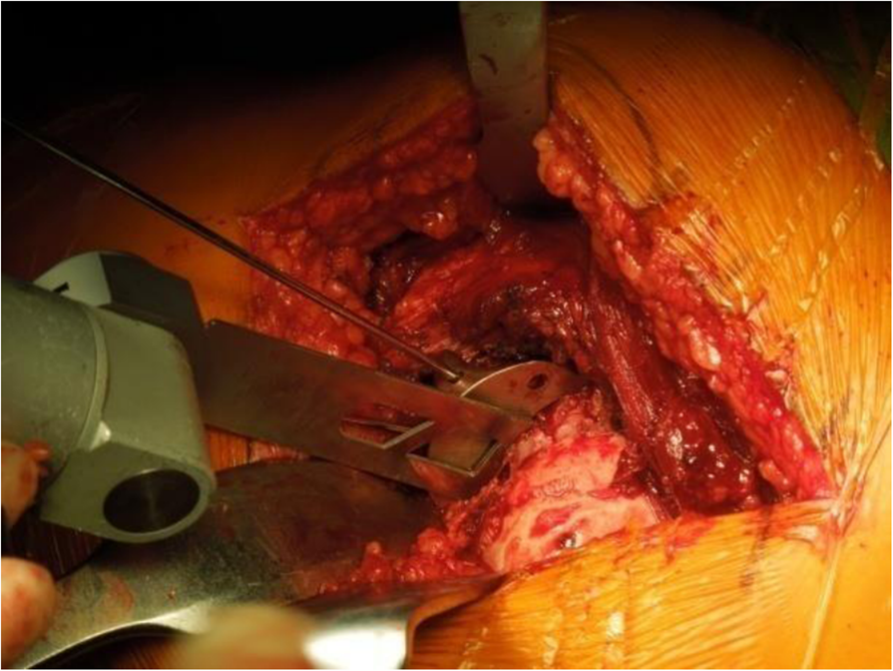
**Before osteotomy: the device is secured on the top of the lesser trochanter by the locating seat and fixed with 2.0 Kirschner pins after adjusting the angle.**

**Figure 4:**
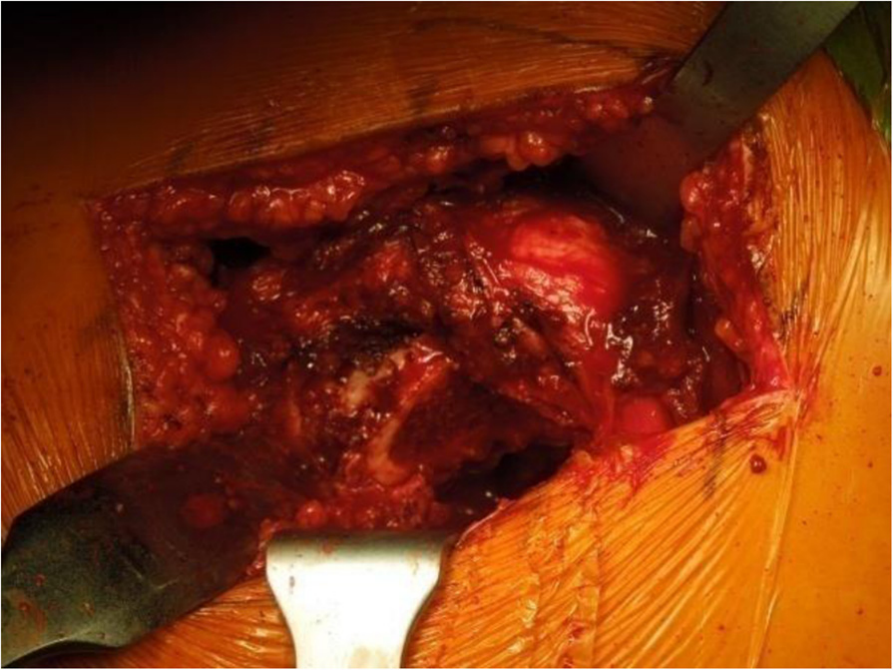
**Showing the platform after osteotomy after the guide has been removed.**

The operations were performed through a posterolateral approach with patients in a lateral decubitus position. After exposing the femoral neck and the lesser trochanter, the surgeon placed the guide on the lesser trochanter. It is important to place the locating seat on the top of the lesser trochanter and to adjust the oriented platform to be perpendicular to the long axis of the femoral neck. The height of the bone resection is determined by the height of the guide, but the angle of the osteotomy was adjusted by lifting or lowering the platform of the guide by eye by the surgeons based on their experience. After fixing the guide with Kirschner pins at a diameter of 2 mm, the guide was fastened to femoral neck, making it unnecessary to hold the device tightly against the bone while performing the resection. Intraoperative X-ray was not performed after positioning the guide in front of the osteotomy. The surgeon cut the femoral neck while the saw was in contact with the guide platform (Figure 3). After the bone was resected, the device was removed to show a smooth bone platform perpendicular to the long axis of the femur (Figure 4). The prosthesis was then placed following the customary surgical steps and post-operative X-rays were taken. For group II, the same procedure was followed, with the exception of using the guide.

The Institutional Review Board of the Third Hospital of Hebei Medical University approved this study after thorough examination and verification. All patients signed informed consent and agreed to participate in the study.

### Statistics

Statistical analysis was carried out by using SPSS13.0 for Windows (SPSS, Chicago, IL, USA). Enumeration data of the two groups of patients organised by gender, aetiologies of indications and operative side were compared by the Chi-squared test. Measurement data, such as age, were compared using a *t*-test. The significance threshold was set at P < 0.05.

## Results

A total of 48 patients participated in the study. Gender, age, operative side and clinical data were collected for statistical analysis, with results showing no significant difference between the two groups (Table 1). Difference existed between the planning and postoperative osteotomy heights in both group, but the mean difference in group I (average, 0.84 mm) was significantly less than group II(average, 1.69 mm) (P < 0.001) (Tables 2 and 3). Patients undergoing THA in group II had an average difference in osteotomy height of 1.69 mm, while group I had an average difference of only 0.84 mm, which was significantly less than in group II (P < 0.001) (Table 4). The guide group had an average postoperative LLD of 5.45 mm, while group II had an average LLD of 13.37 mm. Additionally, limb shortening occurred in patients in group II but this was not observed in any patients in group I (Table 5). For both groups, there was no significant difference in operation time (p = 0.406), but the average time for group I was four minutes shorter than that in group II (Table 6). No complications occurred in either group during the process of osteotomy.

**Table 1:** **Characteristics of the two groups**

	**Group I**	**Group II**	**Statistic**	**P value**	**NS**
Number of patients	24	24			
Male	15	11	*X* ^2^ = 1.343	0.247	*
Female	9	13			
Mean age(yrs)	52.31 (range,22-83)	54.55 (range,25-78)	t = 0.448	0.657	*
Operation side			*X* ^2^ = 2.116	0.146	*
Left	11	16			
Right	13	8			
Aetiology of indications			*X* ^2^ = 1.532	0.655	*
Femoral head necrosis	16	18			
Femoral neck fracture	3	4			
Primary osteoarthritis	5	2			

NS: non-significant. *: P>0.05.

**Table 2:** **Planning and postoperative osteotomy height in group I (mm)**

	**‾X ± S**	**T value P value**
Planning	1.30 ± 0.22	10.126 < 0.001
Postoperative	2.14 ± 0.34

**Table 3:** **Planning and postoperative osteotomy height in group II (mm)**

	**‾X ± S**	**T value P value**
Planning	1.27 ± 0.22	13.196 < 0.001
Postoperative	2.96 ± 0.59

**Table 4:** **Difference in osteotomy height of femoral neck between two groups (mm)**

	**‾X ± S**	**T value P value**
Group I	0.84 ± 0.22	5.567 < 0.001
Group II	1.69 ± 0.72

**Table 5:** **LLD between the two groups (mm)**

	**‾X ± S**	**T value P value**
Group I	5.45 ± 2.23	7.609 < 0.001
Group II	13.37 ± 4.58

**Table 6:** **Difference in operation time between two groups (min)**

	**‾X ± S**	**T value P value**
Group I	125.00 ± 17.45	0.838 0.406
Group II	129.13 ± 15.93	

## Discussion

The study focused on introducing and evaluating the femoral neck osteotomy guide, consisting of the oriented part and locating part. The guide was designed for improving the accuracy of osteotomy during THA surgery through the posterolateral approach. The difference between actual and planning osteotomy heights in the group with the guide was lower than that in the group without the guide. In addition, the guide is easy to use and does not prolong the operation time.

Hip replacement is currently one of the most effective treatments for severely diseased or degenerated hip joints. More than 1 million hip arthroplasties are performed every year worldwide, and this number is projected to double within the next two decades [1]. The incidence of LLD after THA has been reported to range from 1% to 27%, with some reports of even up to 50%. It is difficult to eliminate LLD after THA. Combined use of a preoperative femoral template to predict the necessary length correction and plan the femoral neck osteotomy level along with intraoperative measurements is a practical method for avoiding LLD. However, some studies concluded that a planning match exists in only up to 60% of cases, so improving the accuracy of actual performance according to preoperative plan remains controversial [3,5]. LLD following hip replacement may cause lower back pain, sciatic nerve palsy, gait dysfunction, hip dislocation and prosthetic loosening, as well as increasing patient dissatisfaction. LLD has been a major post-surgical complaint for patients receiving hip arthroplasty [6]. LLD is the result of a complex interaction between bone length, implants, soft tissue contractures, and pelvic obliquities. After THA surgery, some authors feel that over-lengthening of the implant head-neck distance resulted in LLD [7,8]. In addition, the restoration of femoral offset is also critical for avoiding LLD, as increased femoral offset theoretically can lead to increased implant bending moments and early loosening. During the operation, inaccurate abduction/adduction repositioning of the femur with respect to the pelvis also can cause substantial errors in the measurement of length and offset change [9]. A variety of methods have been used to avoid or reduce the incidence of LLD, such as preoperative template measurement or using an L-shaped calliper and other surgical devices [10]. Careful preoperative planning is critical but does not preclude the surgeon from choosing the incorrect components for THA. More importantly, attention to detail both in the planning and performance of the surgery may assist in reducing LLD [11]. Although meticulous preoperative templating combined with X-ray, CT and other imaging systems assists with sizing an appropriate prosthesis and can be helpful for avoiding LLD, surgeons may unintentionally stray from the template when implants of different sizes or offsets are used [2]. Although some instruments were used for measuring during the surgery, no professional surgical tools such as those used in knee replacement surgery exist for femoral neck resection. Surgeons typically rely on their experience and finger measurements during the osteotomy. This may be particularly difficult for inexperienced surgeons, especially when trying to gauge the cuneiform plane angle and height of the femoral neck resection. This can extend the operation time, increase blood loss and complicate the procedure. Earlier studies paid close attention to measurement methods using different types of devices and landmarks, but difficulties with operative manipulation have not been addressed thus far. The guide was invented to address the uncertainty of femoral neck osteotomy. In our study, the surgeons had more than 10 years of experience in hip replacement, yet still had a certain degree of error. In the current study, errors in osteotomy height between the actual and predicted size were reduced to 0 ~ 1 mm. Furthermore, LLD was reduced to less than 10 mm. We believe that such a device will become standard in femoral neck osteotomy, resulting in more precision for cutting height and resection angles. This will also reduce the surgical times and alleviate soft tissue damage during the operation.

Our study has some limitations. First, the head-neck distance of the implants was not recorded or compared between the two groups. A device combined femoral neck osteotomy guide with head-neck distance measurement is being developed. Second, in our study, one observer who was blinded to the design was in charge of measurement, so inter-observer agreement was not considered in the design of the study. This was one of the major limitations of the current study. Finally, the soft tissues above the lesser trochanter could not be stripped clean during surgery, which introduced a measurement error in performing the osteotomy. Additionally, in obese patients, it is difficult to expose the lesser trochanter, so the device cannot accommodate all types of patients in clinical practice.

## Conclusions

The guide device, as an auxiliary tool, assists the surgeon in achieving a more precise femoral neck osteotomy according to the preoperative plan, which may also reduce the incidence of LLD. The design still has some limitations, and there is more room for improvement. First, the guide with its fixed height is not convenient in clinical application. If the height of device was adjustable, the surgeon could alter the osteotomy height without removing the location seat. This problem could be solved by adding a gasket on the platform of the device; we are currently working on that modification. Second, the adjustment of the angles in bone resection still depends on the surgeon’s experience; this is another area that needs improvement. The design will be further refined to improve the tool’s functionality.
